# Therapeutics and Neurological Therapeutics

**Published:** 1886-06-20

**Authors:** C. L. Dana


### THERAPEUTICS AND NEUROLOGICAL THERAPUETICS.

BY C. L. DANA, M. D.

[Read at a meeting of the Clinical Society of the Post-Graduate Medical School, introductory to a discussion of Therapeutics.]

In opening a course of lectures recently upon Therapeutics, Prof. George Hayem, of Paris, announced a new classification of remedies. They may, he said, be arranged in two divisions; the *anti-microbic* and the *nutrition* remedies. Such a classification is attractively simple, and shows how the microbe has taken hold upon the medical mind; but it is hardly to be justified in the present state of knowledge, for in Pathology we have not yet been able to say what is the exact function of micro-organisms, and in Therapeutics the attempts to treat disease by germicides have so far been failures. The parasite ofphthisis or of pneumonia, for example, nestled away among the pulmonary tissues, is as safe from the attacks of man as if it were locked in the ooze of a fathomless sea. There are, in fact, no anti-microbic remedies. In preventive midicine it is different. Here germicidal measures are everything; and possibly we may in time learn how to destroy those parasites while they are at work in the body; in this direction there certainly lies a grand field for theraupeutic effort.

But in recent years, it is among "nutrition-remedies," so-called, that any therapeutic advances have been made.

I do not propose to give any general review of these, but beg to refer to two points which have a bearing on the proposed discussion: the first is the importance of keeping in mind the difference between a therapeutical contribution and therapeutical progress. Many a physician believes that he can make, or has made, some valuable therapeutical discovery worthy of being laid before the world. The result is that our literature is loaded with the inconsequential results of poor observation, shallow reasoning, and hasty conclusions. And this is, I fear, particularly the case with American medical literature, which reflects the great American erythism for "something practical." The truth is that there are few harder things to do than to establish upon incontrovertable grounds the fact that a certain remedy relieves a certain pathological condition. Most of our therapeutical work is therefore a kind of scientific frolic between new drugs and old diseases ; as witness the contest that is now going on in this city between the infective fevers and imported German and pyretics. Not that all this is to be deprecated entirely ; for therapeutical triumphs, when they do come, are almost as likely to be the result of some happy chance as of scientific preconceptions. But we must beware of the man who is constantly discovering new remedies. He is generally either dishonest or self-deceived. He is, in either case, sure to be very loose-jointed in his mental make-up.

The best influence brought to bear on modern therapeutics is physiological experimentation. This has not furnished many valuable remedies ; perhaps it never will ; but it has brought into therapeutics a more scientific spirit ; it has taught clinicians to be more careful in observation, and yet has given them greater certainty regarding conclusions reached.

There are three ways now by which we can advance to therapeutical certainty : by the sick-room, where the clinician observes; by the physiologist's laboratory, and, last and not too often, by the dead-house, where we learn our failures.

### PROGRESS IN NEUROLOGICAL THERAPEUTICS.

The incompleteness of medical or surgical specialty is indicated, in a measure, by the variety and novelty of its therapeutical armamentarium. Judged by this test, I fear neurology would have to suffer in scientific estimation. It would hardly be fair, however, to apply that term rigidly here. Only ophthalmologists treat eye diseases of the severer types, but every doctor treats nervous diseases and contributes his mite to its therapeutics. Besides, there has certainly of late years developed a greater uniformity in the treatment of some of the neuroses and organic nervous affections.

In general, I may say that the drift of neurological therapeutics has been towards the more extended and careful use of mechanical agencies, such as electricity, hydrotherapy, massage, nerve-surgery, etc. ; and to a better appreciation of the humoral element in the causation of nervous symptoms.

So far as drugs are concerned, advances have been made in the knowledge of how to use old remedies rather than in the discovery of those that are new.

If I were to enumerate those things which constitute real progress in neurological therapeutics, I should say that they were :

1. The better knowledge of how to use electricity.

2. The better knowledge of how to use the bromides, the iodides, and remedies for metabolic disturbance.

3. The introduction of nerve-stretching and the advances in nerve and cerebral surgery.

4. The Rest-cure, taken in its largest sense.

Among the minor additions I would place—

(1) The introduction of amyl nitrite and its allies, and of hyoscyamine and paraldehyde.

(2) Nerve-vibration.

(3) The wider use of hydrotherapy, and the better application of the rules of diet and right living.

To take up some of the foregoing in detail:

1st. One of the most important additions of late to neurological therapeutics has been the introduction of absolute galvanometers. By means of these one can dose electricity accurately. I have observed that with the same number of cells I can get a tenfold increase in the current strength of electricity by the variations in the manipulations of the electrodes. The old method of measuring electricity is comparable, therefore, to measuring medicines with an elastic spoon that would hold anywhere between ʒ i and ʒ x.

Good milleamperemeters are now made in this city; they cost only $15 to $25, and their method of use is perfectly simple. Barrett's is one of the best.

The introduction of large electrodes is another advantage in electro-therapeutics. With them one can give stronger and more penetrating currents without discomfort to the patients. Unfortunately, many of our electrical instrument-makers do not yet supply proper ones, except on special order.

Using the milleampere of DeWatteville, the electrode, time and electrode measurements of Schular, and the further terms which I would suggest here, one may prescribe galvanism accurately in a formula; for example, the following:

℞ 10 m. a. $^{12}_{25}$ daily. P. stabile. < >

Which means that a current strength of 10 milleamperes is to be used daily with an electrode of a surface of 25 square millemeters; duration of each sitting being 12 minutes, polar method, stabile current, gradually increased, then decreased.

I am confident that static electricity is of real help in some cases in which the other forms of electricity would be extremely inconvenient or useless.

In the use of bromides, we have found that results can be obtained by large doses not obtainable by small, and that bromidization may do what simply giving bromides will not. Slight and minor advantages are gotten by combining and varying bromides. A few of the bromides have a somewhat peculiar effect. Thus hydrobromic acid is almost a specific in many vertiginous and vasomotor troubles. In combining bromides with camphor, zinc, strychine, arsenic, gold, etc., one gets mainly the effect of these latter drugs. Bromides, it is found, can be given in large continuous doses, provided tonics and tonic measures be combined; for example, I often order bromides combined with arsenic, hypophosphites, iron, digitalis, cod-liver oil, coca wine, cold sponging, etc. In this way an epileptic patient can often carry suppressive doses more comfortably.

The use of iodides in large doses has become a familiar one, and is so frequently illustrated in our clinics that I need say nothing of it.

It is one of the few drugs that, when properly given, will sometimes produce marked amelioration in tabes dorsalis.

The efficient dose of strychnine in many cases should be one-fourth to one-eighth of a grain daily.

Hyocyamine (Merck's crystallized), in doses of grain 1-60 to grain $\frac{1}{4}$, has established itself as perhaps the best single remedy in paralysis agitans. It answers best in the early stages of that disease. As an ordinary hypnotic in insomnia it is not perfectly trustworthy. In shortening and ameliorating maniacal attacks it is of unquestionable service, but it must often be given in large doses, and it acts better given hypodermically.

Nitrite of amyl, nitroglycerine and nitrite of sodium have won a place in therapeutics, particularly in the treatment of spasmodic vaso-motor troubles. All three of these drugs act in a similar way, and all have but a temporary physiological effect. One feels the flushing and congestion of nitroglycerine for about half an hour. Given regularly, it in time causes a frontal headache. Nitrite of sodium is said to be a little slower in action than its two congeners. These drugs are of very little use in epilepsy, if we except the amyl, which is of service in warding off attacks. In certain conditions characterized by excessive coldness of the hands and feet glonoin is said to have a good effect, but it must be given often.

Nerve-stretching, in the five or six hundred reported cases, has been proved a reasonably efficacious measure. It has cured, or very greatly ameliorated, about two-thirds of the peripheral nervous diseases for which it has been tried, such as neuralgia and spasms. It has failed in about 8 per cent. of these cases, and caused death in about 5 per cent. On the other hand, it has cured only 3 per cent. of the cases of cerebro-spinal nervous disease ; it has produced temporary amelioration in about 66 per cent., and death in 8 per cent.

Among drugs or therapeutic measures of minor or not well established value, I would place—

Nerve-vibration.
Paraldehyde.
Cannabum Tannicum.
Ligation of vertebral arteries.
Pilocarpin.
Hypnotism.
Chloride of Gold and Potassium, Cyanide of Gold.
Homatropine.

Nerve-vibration, a measure introduced by Dr. Granville, has been employed to some extent by physicians of this city. I have myself had an instrument constructed, and used it in a few cases. It will produce physiological and therapeutical effects upon the nerves to which it is applied, but it appears to me to be neither a very powerful or a very serious therapeutic measure. The vibrations of a tuning-fork have been used by French physicians in treating neuralgias.

Paraldehyde is a moderately good and undoubtedly safe hypnotic, but it is an unspeakably nasty drug to take, and I do not believe it can ever be extensively used, outside of hospitals. In the nervous clinic at the Post-Graduate School it has been sometimes disappearing. Dr. Hammond tells me that he uses it a great deal and with good success. He gives it in teaspoonful doses in sugar.

Urethran I have tried in two or three cases, but cannot yet speak of its value.

Cannabum Tannicum is a fairly good hypnotic in doses of gr. v to gr. x, but it is very expensive.

Homatropine has been successfully used in our department of the school by Dr. Hammond, in the headache due to eye-strain.

The Chloride of Gold and Potassium has been strongly recommended by Professor Bartholow in the melancholia of the aged, in spinal and other scleroses, in sexual hypochondriasis, and in spasmodic troubles. In my experience, gold is not so efficacious in the treatment of tabes as is silver, or the iodides and ergot. Recently I have been using cyanide of gold in the optic atrophy of beginning tabes, as recommended by Galcozowsky. There was a cessation for some months in the previously progressive character of the disease.

Hypnotism has been used in the treatment of neuralgic, spasmodic, and hysterical troubles. Such men as O. Berger, Preyer, DeGiovant, have employed it successfully. In the clinics at our school, Dr. Charles Henry Brown has relieved a number of hysterical troubles by hypnotizing the patients. I would myself never use it, and strongly discountenance its practice except in rare instances ; for hypnotization has, I believe, sometimes a bad effect upon the nervous system. It certainly inculcates a habit of dethronement of the will.

Among the other more or less new things in neurological therapeutics are—

Tincture of prethrum, recommended in globus, by Roth.

Chloride of Methyl, a gas compressed and driven against the limb in sciatica. It freezes the tissues and cures the case, according to Debove. The measure has been tried with much less success by physicians in Lyons and Switzerland. The gas is not obtainable in this city.

Ice-water injections over the nerve in sciatica are recommended by Dr. S. Pollak.

Apomorphine, in nauseating doses, has been used in hystero-epilepsy. I have found it very effective in breaking up hysterical attacks.

Magnets. These have been proved, by Bourneville and Bricon, to have no value in epilepsy, but they appear to relieve some forms of sensory paralysis and hysterical contractures.

Curare is a substance which is, I believe, of no value in epilepsy or in medicine. It is difficult to get it in a pure state, and it must be given freshly prepared ; hence the practical difficulties in its use are great. Bourneville and Bricon have recently shown its inutility in epilepsy. Its reputation rests almost entirely on a doubtful case of cure of rabies, and Kunz's cases of epilepsy.

Moist sculptor's clay laid on over the chest in angina pectoris is recommended by Dr. Masalitinoff.

Croton-chloral, if of value, must be given in large doses, gr. x–xx, or even ʒ i. I cannot speak for its efficacy.

Bromide of arsenic is a therapeutical humbug, since while masking under the form of a bromide it is really only arsenic, giving the same physiological and therapeutical effects.

Veratria in tremor is another failure, according to my experience, and I am corroborated by that of Dr. Hammond, who tells me that he has found it to do no good, but even harm. It was recommended first by Dr. Feres, of Brest, in doses of one-half a millegramme.

Jamaica dogwood, tonga, celery, hydrobromate of conia are all, I fear, disappointing drugs, but I refrain from giving a positive opinion upon them.—*Quarterly Bulletin of the Clinical Society.*

